# Serious Illness Communication in Cancer Care in Africa: A Scoping Review of Empirical Research

**DOI:** 10.1200/GO.22.53000

**Published:** 2022-05-05

**Authors:** Chiara A. Wabl, Raymond Athanas, Vincent Cubaka, Beatrice Mushi, Mamsau Ngoma, Nicaise Nsabimana, Godfrey Sama, Hubert Tuyishime, Pacifique Uwamahoro, Justin J. Sanders, Rebecca L. Sudore, Katherine Van Loon, Evans Whitaker, Rebecca J. DeBoer

**Affiliations:** ^1^ University of California, San Francisco, CA; ^2^ Muhimbili University of Health and Allied Sciences, Dar es Salaam, Tanzania; ^3^ Partners in Health/Inshuti Mu Buzima, Kigali, Rwanda; ^4^ Ocean Road Cancer Institute, Dar es Salaam, Tanzania; ^5^ Partners in Health, Boston, MA; ^6^ McGill University, Montreal, Quebec, Canada

## Abstract

Serious illness communication (SIC) in cancer care describes conversations between clinicians, patients, and families about prognosis and treatment decisions. Cultural context influences SIC. Researchers have studied SIC across diverse settings in Africa. We aimed to describe and synthesize the heterogeneous body of research on SIC practices, preferences, and needs in Africa to identify research and training priorities.

**METHODS:**

Our search strategy identified studies that focused on SIC within cancer or palliative care in Africa. Following PRISMA guidelines, a systematic literature search was performed using PubMed, Embase, Web of Science, CINAHL, African Index Medicus, and PsycINFO, yielding 1811 unique titles. After sequential review of abstracts, full text, and cited references, 42 articles met inclusion criteria. Quantitative and qualitative data describing study characteristics, aims, methods, and findings were abstracted and analyzed using descriptive statistics and thematic analysis. Critical appraisal was performed using the Mixed Methods Appraisal Tool.

**RESULTS:**

The 42 included articles were published from 1997-2021, half since 2017, representing 16 countries and all African Union regions: West (33%), East (29%), South (21%), North (12%), and Central (5%). Most study designs were qualitative (45%) or quantitative surveys (50%). Study participants included patients (35%), family caregivers (18%), doctors (18%), nurses (12%), and/or other (11%). Study aims focused on disclosure of diagnosis (27%) or prognosis (20%), breaking bad news (15%), general patient-clinician communication (12%), truth-telling (8%), shared decision-making (7%), information needs/preferences (5%), and/or advance care planning (5%). Despite diverse contexts, common themes emerged. Study authors frequently recommended communication skills training. Critical appraisal demonstrated high quality of studies overall.

**CONCLUSION:**

Research on SIC in Africa has increased in recent years. Most studies have focused on information delivery by clinicians; fewer on eliciting information from patients (eg, shared decision-making, advanced care planning). Significant opportunities exist for further study and for communication skills training.

